# Microbial Communities of Lycaenid Butterflies Do Not Correlate with Larval Diet

**DOI:** 10.3389/fmicb.2016.01920

**Published:** 2016-11-30

**Authors:** Melissa R. L. Whitaker, Shayla Salzman, Jon Sanders, Martin Kaltenpoth, Naomi E. Pierce

**Affiliations:** ^1^Department of Organismic and Evolutionary Biology, Museum of Comparative Zoology, Harvard University, CambridgeMA, USA; ^2^Insect Symbiosis Research Group, Max Planck Institute for Chemical EcologyJena, Germany; ^3^Department of Pediatrics, University of California San Diego, La JollaCA, USA; ^4^Department for Evolutionary Ecology, Johannes Gutenberg UniversityMainz, Germany

**Keywords:** Lycaenidae, Lepidoptera, gut microbiome, horizontal gene transfer, herbivory, aphytophagy

## Abstract

Herbivores possess many counteradaptations to plant defenses, and a growing body of research describes the role of symbiotic gut bacteria in mediating herbivorous diets among insects. However, persistent bacterial symbioses have not been found in Lepidoptera, despite the fact that perhaps 99% of the species in this order are herbivorous. We surveyed bacterial communities in the guts of larvae from 31 species of lycaenid butterflies whose caterpillars had diets ranging from obligate carnivory to strict herbivory. Contrary to our expectations, we found that the bacterial communities of carnivorous and herbivorous caterpillars do not differ in richness, diversity, or composition. Many of the observed bacterial genera are commonly found in soil and plant surfaces, and we detected known homopteran endosymbionts in the guts of homopterophagous species, suggesting that larvae acquire gut bacteria from their food and environment. These results indicate that lycaenid butterflies do not rely on specific bacterial symbioses to mediate their diverse diets, and provide further evidence of taxonomically depauperate bacterial communities among Lepidoptera.

## Introduction

Plants present numerous nutritional and defensive challenges to potential herbivores, and a large and growing body of research emphasizes the role of symbiotic microbes in ameliorating herbivorous diets. The argument that symbiotic bacteria can facilitate herbivory among insects is not new (Berenbaum, 1988; Barbosa et al., 1991; Felton and Tumlinson, 2008; Feldhaar, 2011), but with advances in high-throughput sequencing technologies, it has become increasingly feasible to describe the bacterial communities associated with insects and identify the contributions that symbiotic bacteria make to host nutrition.

Bacterial genomes encode vastly more diverse metabolic capabilities than eukaryotic genomes (Lapierre and Gogarten, 2009), such that symbiotic gut bacteria can profoundly expand the metabolic repertoire of their insect hosts. For example, bacteria can help insects to degrade indigestible plant fibers: bacterially mediated cellulose degradation has been shown in beetles (Bayon and Mathelin, 1980; Vazques-Arista et al., 1997), and the guts of bees are known to harbor bacteria that aid in the degradation of pectin found in pollen (Engel et al., 2012). Some bacteria are capable of detoxifying plant secondary compounds such as phenolics, glucosinolates, and some alkaloids (Hammer and Bowers, 2015), thereby enabling their insect hosts to feed on chemically defended plants (e.g., Boone et al., 2013). Other bacteria are capable of synthesizing essential amino acids (Baumann, 2005) and vitamins that are unavailable from plant tissues and which insects are incapable of synthesizing on their own (Salem et al., 2014). One of the most striking examples of insect-bacterial nutritional symbiosis is the well-known association between aphids and their endosymbiont *Buchnera* (Douglas, 1998), with similar obligate associations in other sap-feeding Hemiptera. Given that bacterial symbionts can so profoundly influence the diets of their insect hosts, it is not surprising that many nutritional partnerships between herbivorous insects and their gut bacteria have led to host plant specialization and expansion (Tsuchida et al., 2004; Janson et al., 2008; Sudakaran et al., 2015). The gut microbiomes of diverse groups of insects are now recognized as key factors in shaping and promoting ecological diversification (Hammer and Bowers, 2015).

Persistent bacterial associations are known in several major insect orders, including the Coleoptera (Campbell et al., 1992; Scott et al., 2008; Ceja-Navarro et al., 2015), Hemiptera (Buchner, 1965; Douglas, 1998; McCutcheon and von Dohlen, 2011; Salem et al., 2012), Hymenoptera (Kaltenpoth et al., 2005; Russell et al., 2009b; Martinson et al., 2011; Sanders et al., 2014), Diptera (Behar et al., 2005; Shin et al., 2011; Wang et al., 2013), and Blattodea (Potrikus and Breznak, 1981; Brune, 2014; Hongoh, 2014). However, they have yet to be described in detail for Lepidoptera. The Lepidoptera form one of the largest insect orders and include many economically important species that act as agricultural pests, conservation targets, pollinators, and biological models. They are also a largely herbivorous group, with the overwhelming majority of species feeding on plants (Strong et al., 1984).

The prevalence of herbivorous diets makes Lepidoptera good candidates for investigating potential bacterial contributions to mediating the challenges of herbivory. Studies of lepidopteran gut microbiomes have focused largely on pest species and laboratory models (e.g., Broderick et al., 2004; Brinkmann et al., 2008; Robinson et al., 2010; Belda et al., 2011; Tang et al., 2012). These studies have found relatively simple, often transient bacterial communities, suggesting that specialized bacterial associations may not be as important for Lepidoptera as they are in other insect groups. However, most of these studies focus on a single species reared under laboratory conditions, often on artificial media (but see Hammer et al., 2014; Staudacher et al., 2016). While other studies have looked at bacterial shifts in response to different host plant use (Priya et al., 2012; Tang et al., 2012; Landry et al., 2015) or in inducing host plant shifts (Tsuchida et al., 2004), none have explored the role of bacterial communities in mediating major dietary transitions within the group.

We conducted a comparative study of the gut microbiomes of carnivorous and herbivorous Lepidoptera to provide context to the role of the microbiome in facilitating major transitions to and from herbivorous diets. Aphytophagy has arisen multiple times within the Lepidoptera: it is known from at least 14 families of moths, and in particular, has arisen many times within the butterfly family Lycaenidae, most likely as a result of the close associations between lycaenid larvae and ants (Pierce, 1995). The diversity of feeding ecologies makes the Lycaenidae an ideal system to look into a potential microbial association with diet in the Lepidoptera.

Most lycaenid species associate with ants during their larval stages. Caterpillars produce nutritious secretions from specialized exocrine glands to reward and appease ants in exchange for ants’ protective services. Ant-lycaenid interactions are diverse, and although the majority of these associations are considered mutualistic, a number of species have switched to parasitic lifestyles in which caterpillars enter their host ants’ nests and feed either on ant regurgitations (trophallaxis) or directly on ant brood. Other species indirectly parasitize their host ants by feeding on ant-tended insects such as aphids, mealybugs, and planthoppers (Fiedler, 1991; Pierce et al., 2002). Thus lycaenid diets range from phytophagy on specialized host plants to strict aphytophagy on ants and ant-associated insects. Whereas lycaenids comprise a relatively small number of lepidopteran species, they represent a disproportionate number of carnivorous Lepidoptera (Pierce, 1995), and herbivorous and carnivorous species are dispersed across the group, with at least 13 independent origins of carnivory within otherwise herbivorous clades (Pierce et al., 2002).

Given the unusual diversity of feeding ecologies among lycaenids, it follows that larvae with different diets might require different assemblages of bacteria in their guts to modulate their extreme diets. Herbivorous species may harbor bacteria that assist with degrading plant cell walls, detoxifying plant allelochemicals, or provisioning nitrogen or other nutrients that are unavailable from their host plants. Conversely, maintaining associations with these same gut microbes would not be beneficial for carnivorous species that experience a different suite of dietary challenges. Instead, carnivorous species may require a gut community that is effective in fatty acid or protein metabolism, or degrading the chitinous exoskeletons of their insect prey.

Earlier surveys of the heritable bacteria associated with adult lycaenid butterflies and ants (Russell et al., 2009a, 2012) found that lycaenids harbor only two vertically transmitted symbionts, *Wolbachia* and *Spiroplasma* – and *Wolbachia* was by far the most dominant heritable symbiont found in association with the species surveyed. *Wolbachia* are known reproductive manipulators in many Lepidoptera (Hurst et al., 1999; Jiggins et al., 2000; Hiroki et al., 2002), though recent work has shown that *Wolbachia* can provide a defensive advantage in some insects (Hedges et al., 2008; Hamilton and Perlman, 2013; Becerra et al., 2015), and even act as a nutritional mutualist in others (Brownlie et al., 2009; Hosokawa et al., 2010; Nikoh et al., 2014), raising the possibility that *Wolbachia* may be important symbionts in lycaenid larvae as well.

In this study, we investigated the gut bacterial communities associated with wild-caught carnivorous Lycaenidae and their herbivorous relatives. We sampled broadly across the Lycaenidae to identify potential convergence in the bacterial communities associated with species with similar larval diets.

## Materials and Methods

### Sample Collection

Samples were collected in 2014 and 2015 in the United States, Kenya, South Africa, Singapore, Australia, and Denmark. Lycaenid larvae that were third instar or older were located by surveying known host plants and excavating ant nests. Larvae were collected with sterile forceps and placed in a sterile dish with no food or ants for a minimum of 5 h to allow their guts to evacuate. After this fasting period, larvae were killed in ethanol, rinsed in sterile PBS, and their guts were dissected using flame-sterilized dissecting tools. Dissected guts and frass were preserved in separate vials containing 97% ethanol. For comparison, some larvae were not starved or dissected but were processed whole; these samples were rinsed in 10% bleach solution prior to DNA extraction. See **Table 1** for detailed sample information.

**Table 1:** Sample information.

Species	Diet	Food	Country	F/G/W	Subfamily	Tribe
*Trimenia argyroplaga*	Herbivorous	Unknown	South Africa	–/1/–	Lycaeninae	Aphnaeini
*Aloeides pallida*	Carnivorous	Ant Brood	South Africa	–/2/–	Lycaeninae	Aphnaeini
*Aloeides thyra*	Herbivorous	*Zygophyllum*	South Africa	2/5/–	Lycaeninae	Aphnaeini
*Crudaria wykehami*	Carnivorous	Trophallaxis	South Africa	–/11/2	Lycaeninae	Aphnaeini
*Chrysoritis thysbe*	Herbivorous	*Zygophyllum*	South Africa	3/4/–	Lycaeninae	Aphnaeini
*Chrysoritis chrysanta*	Herbivorous	Not recorded	South Africa	1/1/–	Lycaeninae	Aphnaeini
*Chrysoritis perseus*	Herbivorous	*Zygophyllum*	South Africa	1/1/–	Lycaeninae	Aphnaeini
*Lycaena clarki*	Herbivorous	*Rumex*	South Africa	–/3/–	Lycaeninae	Lycaenini
*Anthene usamba*	Herbivorous	*Vachellia*	Kenya	5/16/3	Lycaeninae	Polyommatini
*Anthene* sp.	Herbivorous	*Acacia*	South Africa	–/–/2	Lycaeninae	Polyommatini
*Chilades pandava*	Herbivorous	*Cycas*	Singapore	1/7/–	Lycaeninae	Polyommatini
*Maculinea alcon*	Carnivorous	Trophallaxis	Denmark	–/6/5	Lycaeninae	Polyommatini
*Maculinea alcon*	Herbivorous	*Gentiana* Flowers	Denmark	–/4/5	Lycaeninae	Polyommatini
*Lachnocnema bibulus*	Carnivorous	Homoptera	South Africa	–/4/1	Miletinae	Lachnocnemini
*Thestor yldizae*	Carnivorous	Ant Brood	South Africa	–/11/–	Miletinae	Lachnocnemini
*Logania marmorata*	Carnivorous	Homoptera	Singapore	–/7/–	Miletinae	Miletini
*Miletus bigsii*	Carnivorous	Homoptera	Singapore	2/3/1	Miletinae	Miletini
*Feniseca tarquinius*	Carnivorous	Homoptera	USA	2/3/4	Miletinae	Spalgini
*Spalgis epius*	Carnivorous	Homoptera	Singapore	2/5/1	Miletinae	Spalgini
*Durbania amakosa*	Herbivorous	Lichen	South Africa	1/7/2	Poritiinae	Pentilini
*Eumaeus atala*	Herbivorous	*Zamia*	USA	–/–/6	Theclinae	Eumaeini
*Strymon melinus*	Herbivorous	Not recorded	USA	–/–/1	Theclinae	Eumaeini
*Eooxylides tharis*	Herbivorous	*Smilax*	Singapore	–/1/–	Theclinae	Theclini
*Iolaus mimosae*	Herbivorous	Mistletoe	South Africa	–/1/–	Theclinae	Theclini
*Iolaus trimeni*	Herbivorous	*Tapinanthus*	South Africa	–/1/–	Theclinae	Theclini
*Leptomyrina* sp.	Herbivorous	*Cotyledon*	South Africa	–/1/1	Theclinae	Theclini
*Rapala iarbus*	Herbivorous	*Melastoma*	Singapore	–/1/–	Theclinae	Theclini
*Surendra vivarna*	Herbivorous	*Albizia*	Singapore	1/2/–	Theclinae	Theclini
*Flos* sp.	Herbivorous	Not recorded	Singapore	–/1/–	Theclinae	Theclini
*Flos apidanus*	Herbivorous	*Syzygium*	Singapore	–/6/–	Theclinae	Theclini
*Jalmenus evagoras*	Herbivorous	*Acacia*	Australia	–/–/16	Theclinae	Zesiini
*Jalmenus daemeli*	Herbivorous	*Acacia*	Australia	–/–/4	Theclinae	Zesiini


*Larval food source is given as the host plant genus (if known) for herbivorous species, and insect food for carnivorous species. The number of replicates per species is given as the number of frass (F), dissected gut (G), and whole larvae (W) samples. Tribe designations follow Eliot (1973).*

### DNA Extractions and Sequencing

Samples collected in 2014 and 2015 were processed and sequenced separately, using identical methods. DNA was extracted using the PowerSoil DNA Isolation Kit and protocols provided (MoBio Laboratories, Carlsbad, CA, USA), with the addition of a proteinase-K lysis step prior to cell disruption by bead-beating. Extracted DNA was quantified using a Qubit fluorometer (Invitrogen Inc.) for samples collected in 2014 or a NanoDrop TM1000 spectrophotometer (Thermo Scientific) for 2015 samples, and samples containing low amounts of extracted DNA were concentrated using the isolation kit’s suggested protocol.

Extracted DNA was sent to Argonne National Laboratories (Lemont, IL, USA) for library preparation and sequencing of the V4 region of the 16S rRNA gene. Amplicon libraries were prepared using barcoded primers 515F (5′-GTGYCAGCMGCCGCGGTAA - 3′) and 806R (5′ - GGACTACNVGGGTWTCTAAT - 3′) and previously published methods (Caporaso et al., 2012). Libraries were pooled and sequenced on an Illumina MiSeq sequencer using 150 bp paired-end sequencing technology. Sequences and associated metadata were deposited in the EMBL-EBI database^1^ under accession number ERP019556.

### Data Preprocessing

Sequence libraries were demultiplexed using QIIME version 1.8.0 (Caporaso et al., 2010a) applying a minimum Phred quality score of 20. Because read quality was lower in reverse reads than forward reads, only high quality forward reads were used. The 2014 and 2015 sequencing libraries were concatenated and sequences were *de novo* clustered at 97% identity into Operational Taxonomic Units (OTUs) using UPARSE (Edgar, 2013). Chimeric sequences were removed using UCHIME (Edgar et al., 2011) and the GOLD reference database (Reddy et al., 2015). Taxonomy was assigned to clusters within QIIME with the RDP classifier (Wang et al., 2007) trained on the Greengenes database version 13_8 (DeSantis et al., 2006) with default confidence levels. Representative sequences were then aligned using PyNast (Caporaso et al., 2010b) and a phylogenetic tree was constructed using FastTree (Price et al., 2009) implemented in QIIME.

The resulting biom table, phylogenetic tree, and mapping file were imported into R (R Core Team, 2016) as a phyloseq data object using the phyloseq package (McMurdie and Holmes, 2013). Non-bacterial OTUs (e.g., Archaea and unclassified sequences) were removed, and OTU tables were rarified to 10,000 sequences per sample, retaining samples with at least 1,000 sequences.

Bacterial communities were characterized using the phyloseq package in R. Exploratory analyses demonstrated that the prevalence of chloroplast sequences varied among samples. Due to ancient homology between bacterial 16S and Eukaryotic organellar 16S sequences, it is common for chloroplast and mitochondrial sequences to co-amplify with bacterial sequences when using universal bacterial primers (Ghyselinck et al., 2013; Hanshew et al., 2013). These non-target sequences are typically removed prior to analysis, but because we aimed to compare the gut communities of herbivorous and carnivorous insects, we first examined the prevalence of chloroplast sequences across samples. Proportions of chloroplast to non-chloroplast sequences were calculated for each frass, dissected gut, and whole larva sample. Non-parametric Kruskal–Wallis tests (adjusted for multiple comparisons) and two-sample Wilcoxon rank sum tests were used to compare chloroplast prevalence between tissue types and diets, respectively, using the stats and pgirmess packages (Giraudoux, 2016; R Core Team, 2016). Following these statistical tests, chloroplast and mitochondria sequences were removed.

We also observed highly variable prevalence of *Wolbachia* sequences, with *Wolbachia* comprising over 95% of some samples’ total libraries. Non-parametric tests were again used to compare *Wolbachia* prevalence between tissue types and diets. Because *Wolbachia* are likely to be present intracellularly in the tissues of either the host or prey and are not known to be an important functional contributor extracellularly in the gut (Pietri et al., 2016), these sequences were removed prior to downstream analysis.

After removing these sequences, the OTU table representing gut and larva samples was re-rarefied and filtered of OTUs comprising less than 0.01% of the rarefied library within each species. Observed richness and Shannon diversity measures of herbivorous and carnivorous samples were compared using two-sample Wilcoxon rank sum tests. Weighted and unweighted UniFrac pairwise distance matrices were calculated and visualized using NMDS ordination. Family and genus level taxonomy were summarized in separate OTU tables and subjected to Linear Discriminant Analysis using the Galaxy (Afgan et al., 2016) implementation of LEfSe (Segata et al., 2011) with a minimum logarithmic LDA score of 2 and a maximum *p*-value of 0.05.

### Estimated Functional Profiles

The functional profiles of lycaenids’ gut bacteria were estimated using PICRUSt (Langille et al., 2013) within the Galaxy server. The filtered, rarefied OTU table from R was further filtered to remove *de novo* OTUs and retain only OTUs with Greengene IDs. After normalizing the resulting closed reference OTU table in PICRUSt, the table was used to predict metagenomic functions in the form of KEGG Orthologs (Kanehisa and Goto, 2000), which were then collapsed into KEGG Pathways at levels 2 and 3 in the KEGG Orthology hierarchy. Both tables were tested for the presence of differentially abundant features using LEfSe.

### Enterobacteriaceae Tree

Some of the most commonly observed OTUs in our dataset were identified as belonging to the Enterobacteriaceae, a bacterial family that includes many known insect symbionts. In order to identify the phylogenetic placement of these Enterobacteriaceae OTUs, a phylogenetic tree was constructed within the framework of existing GenBank stored sequences found through NCBI’s MOLE-BLAST tool for clustering sequences with their database neighbors. The resulting sequences and alignment were downloaded, and models of evolution were tested with JModelTest 2.1.10 (Darriba et al., 2012). Using Bayesian Information Criterion, the TPM2+G model was selected with a minus log likelihood of 1322.5509. A maximum likelihood tree was estimated using this model in PhyML 3.1 (Guindon et al., 2010).

## Results

**FIGURE 1:**
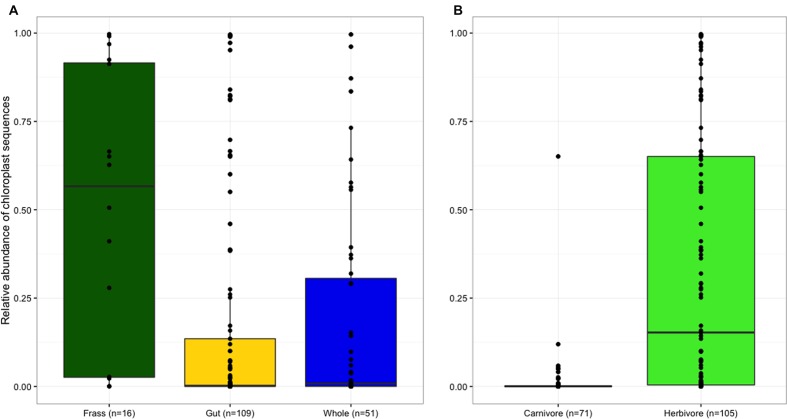
**Frass samples contained significantly higher proportions of chloroplast sequences than both dissected guts and whole larvae, but the proportion of chloroplast sequences in whole larvae and dissected guts did not significantly differ **(A)**.** Chloroplast prevalence was significantly higher in herbivorous samples than carnivorous individuals **(B)**.

**FIGURE 2:**
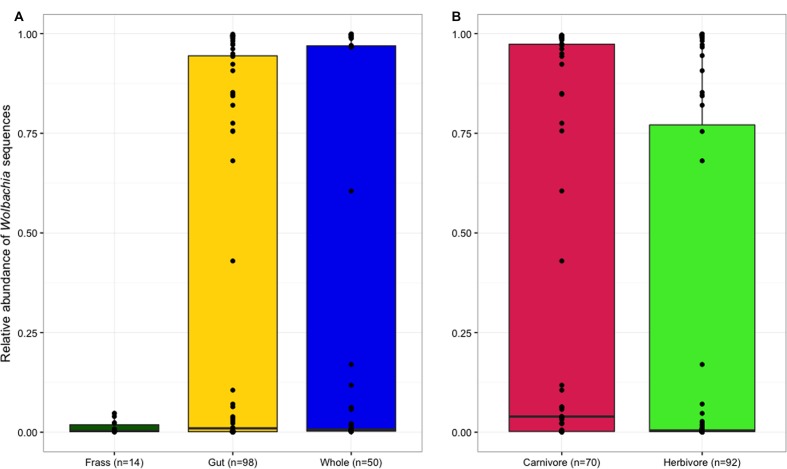
***Wolbachia* sequences were mostly absent from frass samples, but were similarly abundant in dissected guts and whole larvae **(A)**.** *Wolbachia* prevalence was not significantly different between larval diets **(B)**.

Frass samples contained significantly higher proportions of chloroplast sequences than both dissected guts and whole larvae (*p* < 0.05; **Figure 1A**), but the proportion of chloroplast sequences did not differ between samples of whole larvae that had not been starved and samples of dissected guts from larvae that had been starved, suggesting that starvation and dissection were not effective in removing chloroplasts from the gut. As expected, chloroplast prevalence was significantly higher in herbivorous samples than carnivorous individuals (*p* < 0.001, **Figure 1B**). *Wolbachia* was largely absent from frass samples but similarly abundant in both whole larvae and dissected guts (**Figure 2A**), indicating that *Wolbachia* are present in the gut tissues of lycaenid larvae and not simply in other tissues such as reproductive organs and nervous tissue. No significant difference in *Wolbachia* prevalence between diets was observed (**Figure 2B**).

**FIGURE 3:**
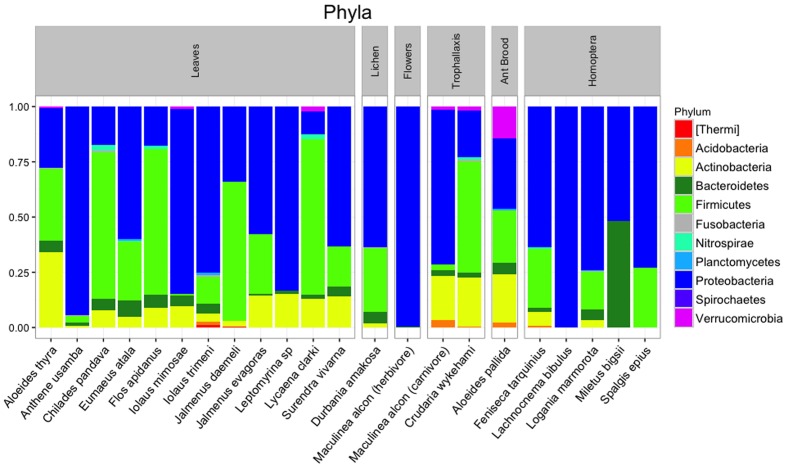
**Bacterial phyla present in lycaenid guts, with relative abundances averaged across individuals of the same species.** Dominant phyla are similar to other animal-associated bacterial communities and include Bacteroidetes, Firmicutes, Proteobacteria, and Actinobacteria.

**FIGURE 4:**
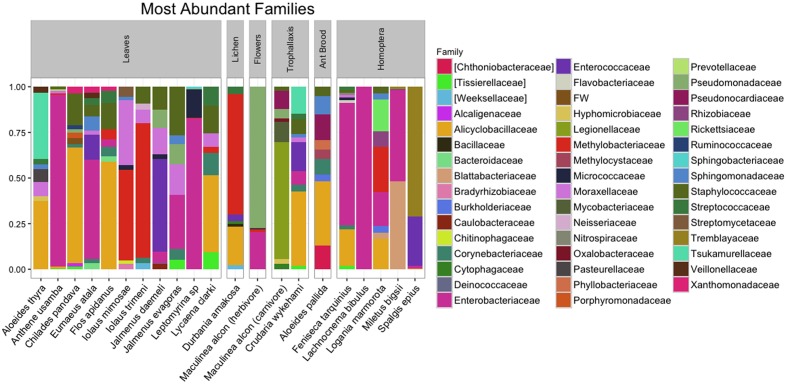
**The 10 most abundant bacterial families present in the guts of lycaenid larvae.** OTU tables were sorted independently for all lycaenid species. The most abundant family, Enterobacteriaceae, includes several known insect symbionts (see **Supplementary Image 1** for the phylogenetic placement of observed Enterobacteriaceae OTUs).

**FIGURE 5:**
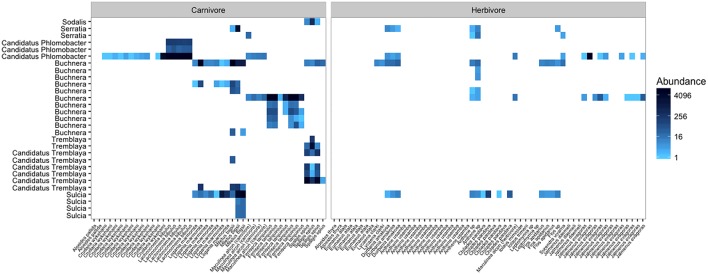
**The abundances of insect symbionts present in carnivorous and herbivorous lycaenid guts.** The color scale is a log transformation with base 4, the default scale for the phyloseq package. Insect symbionts are most abundant in entomophagous lycaenid species, but are present in lower abundances in some herbivores as well. This could be attributable to sequencing errors, or it could be that facultative or accidental homopterophagy is more common than previously thought.

After quality filtering and rarefaction, we identified 1,156 OTUs across 83 lycaenid samples. Dominant phyla included Bacteroidetes, Firmicutes, Proteobacteria, and Actinobacteria (**Figure 3**). Observed OTUs belong to a total of 115 bacterial families, the most abundant of which are shown in **Figure 4**. The 10 most abundant families across the entire dataset were Enterobacteriaceae, Alicyclobacillaceae, Staphylococcaceae, Methylobacteriaceae, Enterococcaceae, Moraxellaceae, Pseudomonadaceae, Corynebacteriaceae, Sphingomonadaceae, and Tremblayaceae. The most abundant family, Enterobacteriaceae, was observed across both herbivorous and carnivorous species (see **Supplementary Image 1** for the phylogenetic placement of Enterobacteriaceae OTUs). This large bacterial family includes many known insect symbionts such as *Buchnera*, *Tremblaya*, and *Serratia*. Several known insect endosymbionts were detected in the guts of the lycaenids surveyed (**Figure 5**), particularly in entomophagous species.

**FIGURE 6:**
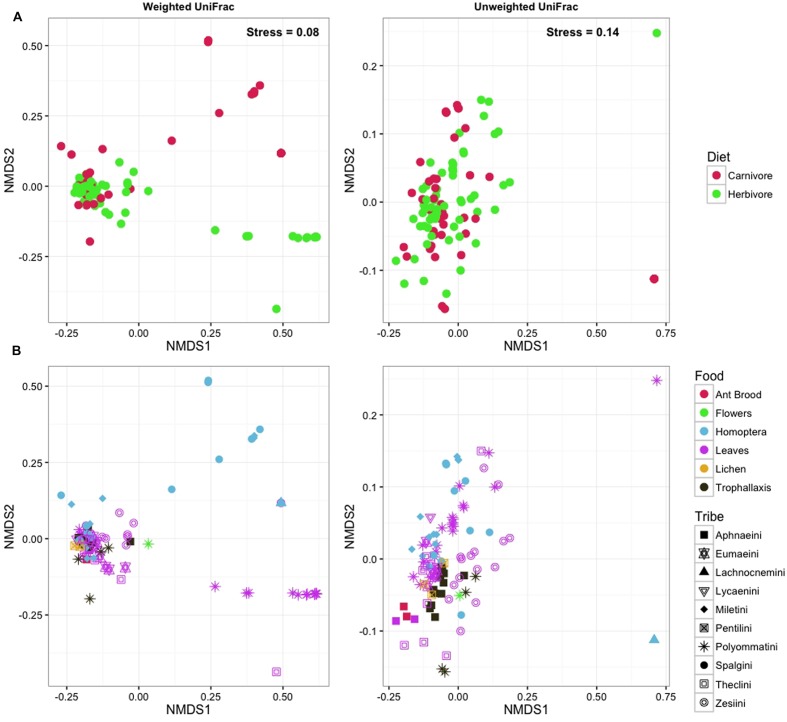
**NMDS plots with points colored according to larval diet** **(A)** and according to lycaenid species with shapes corresponding to lycaenid tribe **(B)** do not demonstrate compositional similarities according to host diet or phylogeny.

Neither OTU richness nor Shannon diversity differed between herbivorous and carnivorous species. Similarly, ordination methods did not reveal any structure or clustering of lycaenid larvae according to larval diet (**Figure 6A**) or lycaenid tribe (**Figure 6B**). Conspecific samples tended to cluster together, indicating that individuals of the same species tend to have more similar bacterial communities compared to other species with similar diets or those belonging to the same tribe.

LEfSe analysis of predicted metagenomes from the PICRUSt pipeline failed to identify any predicted KEGG genes or pathways that were significantly enriched in either diet at either hierarchical level. Similarly, LEfSe analysis of bacterial taxonomy did not identify any differentially abundant OTUs at either the family or genus levels.

## Discussion

We characterized the gut bacterial communities of diverse lycaenid larvae using 16S rRNA amplicon sequencing. We compared the bacterial abundance, richness, composition, and estimated functional profiles of carnivorous and herbivorous species to test for convergence in the microbiomes of species with similar larval diets. Despite our expectation that the gut microbial communities of herbivorous and carnivorous species would differ consistently, we observed a high degree of stochasticity in the microbiomes of lycaenid larvae, with no significant differences between herbivorous and carnivorous species in bacterial richness, diversity, or community composition. This contrasts with patterns that have been observed in vertebrates (Ley et al., 2008; Muegge et al., 2011) and in ants (Russell et al., 2009b). Moreover, we did not observe any major and repeatable differences in bacterial composition, based on weighted and unweighted UniFrac distance metrics. While ordinations indicate that individuals of the same species tend to harbor more similar bacterial communities in their guts, considerable intraspecific variation among individuals was also observed.

The most striking differences between herbivorous and carnivorous lycaenid gut communities arise from ribosomal DNA sequences that are likely food-derived. In our raw dataset, chloroplast SSU rDNA, likely originating from ingested plant material, dominated many of the herbivorous lycaenid gut samples, but was largely absent from the carnivorous species (**Figure 1B**). Chloroplast sequences dominated herbivore frass to an even greater degree, indicating communities of plant-degrading microbes do not colonize the plant material during its passage through the gut. This suggests that sequence-based approaches such as chloroplast barcoding may be a fruitful means of describing the diets of lycaenid species for which larval diets are unknown. Barcoding methods have proved successful in studying the feeding ecologies of many novel taxa (Clare, 2014; De Barba et al., 2014; Gebremedhin et al., 2016), and the analysis of frass samples permits dietary investigation without destructive sampling.

Among carnivorous lycaenids, many of the abundant bacteria detected in the gut are known to be important symbionts in other insects. Often, the presence of these bacteria in the guts of lycaenid larvae could be traced to the larval food source. For example, *Buchnera* sequences were commonly found in the guts of *Feniseca tarquinius*, *Miletus bigsii*, and *Logania marmorata*, three lycaenid species that feed on aphids. The libraries of *Lachnocnema bibulous* larvae, which feed on ant-tended planthoppers, were nearly entirely comprised of sequences identified as Candidatus *Phlomobacter*, a phloem-borne plant pathogen vectored by planthoppers. Other known homopteran symbionts such as *Sulcia*, *Tremblaya*, and *Serratia* were found in the guts of lycaenids that feed on aphids and mealybugs. While the presence of these bacteria is attributable to the entomophagous diets of these lycaenid species, it is unclear whether they could continue to confer nutritional benefits inside the lycaenid gut. Only a few studies have explored the digestion and decay rates of prey-derived bacteria in the guts of predatory insects (e.g., Paula et al., 2015), and further research is needed to examine whether these endosymbionts might continue to provision nutrients inside the larval gut.

Although we find evidence for food-derived bacteria in the guts of carnivorous lycaenid larvae, we do not observe differences in *Wolbachia* abundance between larval diets, suggesting that insect prey do not horizontally transmit *Wolbachia* to their lycaenid predators. The possibility that *Wolbachia* may be horizontally transmitted via feeding on infected prey has been previously suggested (Kittayapong et al., 2003; Sintupachee et al., 2006), and many Hemiptera are known to harbor *Wolbachia* (Augustinos et al., 2011; Hughes et al., 2011). However, horizontal transmission of *Wolbachia* between insects is most successful within closely related insect groups (Russell et al., 2009a), and it may be that lycaenids and their insect prey are too distantly related for such an exchange.

While our results show that known symbionts are unlikely to be directly associated with carnivorous lycaenid larvae, identifying which bacteria are likely to be food-derived in herbivorous species is not straightforward. Many of the major bacterial groups observed in this dataset are commonly isolated from aquatic and terrestrial sources, and are therefore expected to be environmentally derived in both herbivorous and carnivorous species. Alicyclobacillus, Methylobacterium, Acinetobacter, and Agrobacterium represent some of the most abundant bacterial genera in our dataset, and all are common in soil and leaf surfaces (Vorholt, 2012; Kovaleva et al., 2014; Tianli et al., 2014).

The presence of *Pantoea* in several herbivorous lycaenid samples is also of interest. *Pantoea* is a genus within the Enterobacteriaceae that has been recorded from numerous aquatic, terrestrial, and animal sources (Walterson and Stavrinides, 2015). Members of this genus are well-known plant pathogens, and it is possible that their presence in the guts of herbivorous larvae is simply due to feeding on infected plants. On the other hand, *Pantoea* strains have been isolated from the guts of several animals, including other insects. Although the nature of *Pantoea*-host associations is largely unknown, recent studies have demonstrated that *Pantoea* are important mutualistic symbionts in stinkbugs (Duron and Noël, 2016; Hosokawa et al., 2016). The potential for *Pantoea* to act as pathogenic or mutualistic gut bacteria in Lepidopteran larvae remains unexplored.

Taken together, our results suggest that lycaenids do not maintain distinctive gut communities, but are instead characterized by transient and often food-derived bacteria. Although these bacteria may not seem as important for host fitness as do obligate, vertically transmitted bacteria, it is nevertheless possible for environmentally derived OTUs to contribute to host nutrition. It may be that gut bacteria are highly conserved between lycaenid species with similar diets, but that the most influential OTUs are rare and therefore not likely to be identified using the sequencing and statistical techniques implemented here. However, while the possibility for influential but rare gut bacteria cannot be dismissed, nutritional mutualists might be expected to be reasonably common across individuals and species if they perform important functions for their hosts.

Of course, bacterial communities can differ or converge not only in taxonomic composition, but also in functional composition (Burke et al., 2011). We addressed the possibility that the gut communities of lycaenids with similar diets may be functionally convergent, even if not taxonomically similar, by predicting the functional profiles using PICRUSt. However, this method did not detect any differentially abundant functions in our dataset. The accuracy of this method is limited by the quality and availability of appropriate reference databases (Langille et al., 2013). Although lycaenids do not appear to associate with particular bacterial OTUs, they may associate with *functionally* similar bacteria that mediate important aspects of their diet. The community profiling methods we have used do not provide detailed information about bacterial function, and deep metagenomic sequencing will be necessary to definitively characterize and compare the functional profiles of lycaenid gut bacterial communities.

While symbiotic bacteria are known to be important in many insect species, they represent only a portion of the microbial contributions to insect nutrition. Viruses, protists, and fungi could also increase the metabolic potential of their animal hosts, and it is possible that these other microorganisms mediate the exploitation of host diet in ways that have not yet been identified. Relatively few studies have explored this possibility in other Lepidoptera, although recent work has shown that the fungal communities associated with one species of lycaenid butterfly are relatively small and are likely to be environmentally derived (Harrison et al., 2016).

Despite the inordinate dietary diversity among lycaenid butterflies, their gut bacterial communities do not appear to be more selective than other Lepidoptera. Although innumerable studies have identified obligate or persistent bacterial associations in some insect species, the majority of insects harbor low diversity gut microbiomes that are highly variable among species (Colman et al., 2012). These insects do not seem to rely on the nutritional services of symbiotic gut bacteria, yet they still must cope with the challenges and deficiencies of an herbivorous lifestyle, and it is unclear how they do so.

Perhaps the most intriguing possibility is that lycaenids themselves might possess endogenous genes for encoding important digestive enzymes related to herbivory, as has been shown in some termites, beetles, flies, and crickets (Watanabe et al., 1998; Watanabe and Tokuda, 2010; Whiteman et al., 2012). In such cases, the genes in question could be ancestral within insect clades, or they could be recently acquired from microbes through horizontal gene transfer events (Hansen and Moran, 2014; Wybouw et al., 2016). Cases of horizontal gene transfer between microorganisms and animals are becoming increasingly well documented, including within the Lepidoptera (Husnik et al., 2013), and comparative studies indicate that horizontal gene transfer is particularly prevalent in arthropods, relative to other animals (Hotopp et al., 2007; Hotopp, 2011). Although lycaenid larvae do not appear to maintain readily identifiable associations with gut bacteria, they may have acquired key metabolic capabilities through horizontal gene transfer events during ancient bacterial associations. Subsequent losses of these functions following transitions to carnivory could also explain why reversions from carnivory back to herbivory are unknown among lycaenids.

In this study, we provide further evidence that Lepidoptera harbor taxonomically depauperate gut bacterial communities relative to other organisms, even in a clade whose species exhibit unusual trophic diversity. This is in contrast to other insect groups in which gut bacteria are known to play an important role in mediating host diet, and to vertebrates, including mammals, for which the host diet and phylogeny both contribute to bacterial associations. Although host-microbe symbioses are ubiquitous among animals, the nature of these symbioses likely depends on the exact diets, nutritional limitations, and life histories of animal hosts, and our findings demonstrate that it should not be assumed that gut bacteria influence host diet in all taxa.

## Author Contributions

MW and NP conceived of the project. MW and SS conducted the fieldwork and lab work; and MW, SS, and JS analyzed the data with input from MK. All Authors contributed to the preparation of the manuscript.

## Conflict of Interest Statement

The authors declare that the research was conducted in the absence of any commercial or financial relationships that could be construed as a potential conflict of interest.

## Footnotes

1 http://www.ebi.ac.uk/

## Supplementary Material

**IMAGE 1 | The presence of Enterobacteriaceae OTUs found in herbivorous and carnivorous larvae are mapped onto the phylogenetic tree of Enterobacteriaceae, with pie charts representing the percent of lycaenid species within each diet category for which the OTU was present.**
