# Supplementary figures and images for: Microbial Communities of Lycaenid Butterflies Do Not Correlate with Larval Diet
Source: Front Microbiol. 2016 Nov 30;7:1920. doi: 10.3389/fmicb.2016.01920 (PMC5129467; doi:10.3389/fmicb.2016.01920)

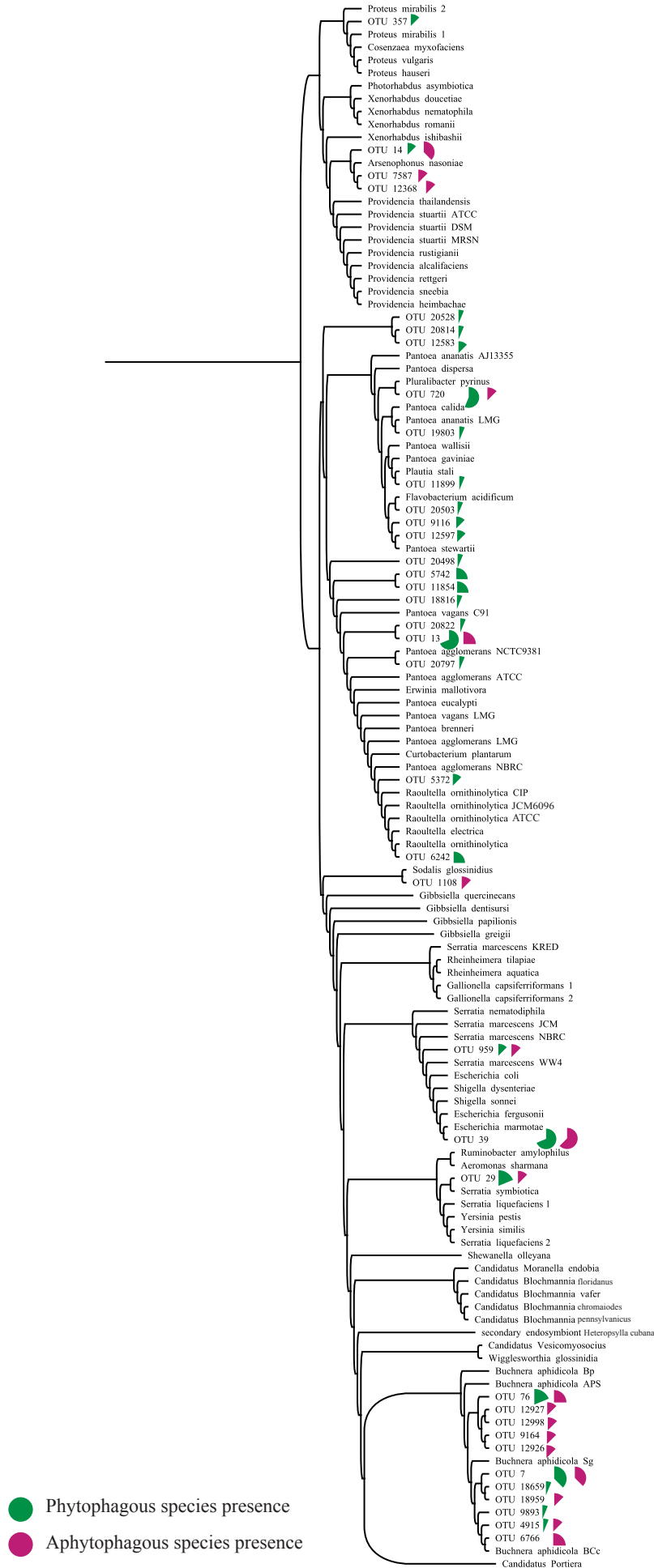

● Phytophagous species presence  
● Aphytrophagous species presence

Supplement: Supplementary file 1 [file Image_1.PDF]
